# Comparative morphology and plant volatile responses of antennal sensilla in *Cinara cedri* (Hemiptera: Lachninae), *Eriosoma lanigerum* (Hemiptera: Eriosomatinae), and *Therioaphis trifolii* (Hemiptera: Calaphidinae)

**DOI:** 10.3389/fncel.2023.1162349

**Published:** 2023-04-27

**Authors:** Lu-Lu Yang, Bing Wang, Jie Shen, Gui-Rong Wang

**Affiliations:** ^1^State Key Laboratory for Biology of Plant Diseases and Insect Pests, Institute of Plant Protection, Chinese Academy of Agricultural Sciences, Beijing, China; ^2^Department of Entomology and Ministry of Agriculture (MOA) Key Laboratory for Monitory and Green Control of Crop Pest, China Agricultural University, Beijing, China; ^3^Shenzhen Branch, Guangdong Laboratory for Lingnan Modern Agriculture, Genome Analysis Laboratory of the Ministry of Agriculture, Agricultural Genomics Institute at Shenzhen, Chinese Academy of Agricultural Sciences, Shenzhen, China

**Keywords:** *Cinara cedri*, *Eriosoma lanigerum*, *Therioaphis trifolii*, primary rhinaria, placoid sensilla, olfactory receptor neuron

## Abstract

Olfaction is important for mediating aphid behaviors and is involved in host location and mating. Antennal primary rhinaria play a key role in the chemoreception of aphids. The function of the peripheral olfactory system in the subfamily Aphidinae has been intensively studied, but little is known about other subfamilies of Aphididae. Therefore, three aphid species were selected to study the olfactory reception of plant volatiles: *Cinara cedri* (Lachninae), *Eriosoma lanigerum* (Eriosomatinae), and *Therioaphis trifolii* (Calaphidinae). In this study, the morphology and distribution of the antennal sensilla of apterous adults were observed by scanning electron microscopy. Three morphological types were identified (placoid sensilla, coeloconic sensilla, and trichoid sensilla); the first two were distributed on the antennal primary rhinaria. A pattern of primary rhinaria in *C. cedri* was found that differed from that of *E. lanigerum* and *T. trifolii* and consists of 1 large placoid sensillum (LP) on the 4th segment, 2 LPs on the 5th segment, and a group of sensilla on the 6th antennal segments. Later, we recorded and compared neuronal responses of the distinct placoid sensilla in the primary rhinaria of the three aphid species to 18 plant volatiles using a single sensillum recording (SSR) technique. The results indicated that the functional profiles based on the tested odorants of the primary rhinaria of the three investigated aphid species were clustered into three classes, and exhibited excitatory responses to certain types of odorants, especially terpenes. In *C. cedri*, the ORNs in LP6 exhibited the highest responses to (±)-citronellal across all tested chemicals, and showed greater sensitivity to (±)-citronellal than to (+)-limonene. ORNs in LP5 were partially responsive to α-pinene and (–)-β-pinene in a dose-dependent manner. Across different species, *E. lanigerum* showed significantly stronger neuronal responses of LP5 to several terpenes, such as (–)-linalool and α-terpineol, compared to other species. In *T. trifolii*, the neuronal activities in LP6 showed a greater response to methyl salicylate as compared to LP5. Overall, our results preliminarily illustrate the functional divergence of ORNs in the primary rhinaria of aphids from three subfamilies of Aphididae and provide a basis for better understanding the mechanism of olfactory recognition in aphids.

## Introduction

Olfaction plays an important role in host location as well as mating in insects (Wechsler and Bhandawat, 2023). The antennae and labial palps are the main appendages on the head for detecting volatile semiochemicals (Wensler, 1974, 1977; Bromley and Anderson, 1982; Pickett et al., 2013). One hypothesis for the olfactory recognition mechanism is as follows: odorants entering the cuticular pore on the sensilla are transported by odorant-binding proteins (OBPs) through the sensillar lymph and activate odorant receptors (ORs) expressed on the dendritic membrane of olfactory receptor neurons (ORNs) (Leal, 2013). The odorants interact with ORs to generate an electrical signal, which is then transmitted as a train of action potentials through the axons of the ORNs to the primary olfactory center of the brain, the antennal lobes (Schmidt and Benton, 2020). Information about the odor is selectively processed by the glomeruli in the antennal lobes and transferred by projection neurons to higher brain centers, including the mushroom bodies and the lateral protocerebrum, which leads to olfactory-guided behaviors (Gadenne et al., 2016).

Insects are sensitive to chemical signals from host plants as well as conspecifics (Nakano et al., 2022). Behaviorally-active semiochemicals have been well-documented in aphids, including alarm pheromones and sex pheromones (Bowers et al., 1972; Dawson et al., 1987a; Zhang et al., 2017). The alarm pheromone component of most Aphidinae species is *trans*-β-Farnesene (EBF) (Francis et al., 2005). Other components, such as germacrene A, isolated from aphids of the genus Therioaphis (Bowers et al., 1977; Nishino et al., 1977), and the monoterpenes [α-pinene, (–)-β-pinene, and (+)-limonene] identified from *Megoura viciae* (Pickett and Griffiths, 1980; Francis et al., 2005), have been shown to function as an alarm pheromone. The sex pheromones of aphid species are mainly composed of nepetalactone and nepetalactol (Dawson et al., 1987a; Birkett and Pickett, 2003), which can effectively attract males (Lilley et al., 1994; Hardie et al., 1997). Iridoid components are other volatiles released from the genus Nepeta (catmints). Therefore, plant-derived nepetalactones can be used to regulate the behavior of male aphids (Birkett and Pickett, 2003). Plant semiochemicals play an important role in aphid host plant location. One study demonstrated that the black bean aphid, *Aphis fabae*, was attracted by the blend released from the healthy broad bean *Vicia faba* L., only one of which, (*Z*)-3-hexen-1-ol, is a main attractant at high concentrations (Webster et al., 2008a,b). Some aphid species specialize in terpenes of plants and use them to locate host-plants. For instance, *Uroleucon nigrotuberculatum*, a common specialist aphid, showed a preference for goldenrod plants containing β-pinene (Williams and Avakian, 2015). On the other hand, volatile compounds emitted by non-host plants have the opposite effect. *A. fabae* is also known to be repelled by isothiocyanates, methyl salicylate, and myrtenal, which are volatile compounds associated with non-host plants (Nottingham et al., 1991; Hardie et al., 1994a). Plants typically modify volatile emissions after aphid feeding. For instance, wheat plants released additional volatile cues, including short-chained alcohols and ketones, to make the plant less attractive for *Rhopalosiphum padi* (Pettersson et al., 1995), suggesting that herbivore-induced volatiles could have repellent effects on aphids.

In aphids, antennae are the main olfactory organs, and are filiform and composed of 5–6 segments, including a scape, a pedicel, and a flagellum with 3–4 flagellomeres (Kanturski et al., 2017; Song et al., 2020; Wu et al., 2022). The main sensory structures are called rhinaria (Wensler, 1974, 1977; Bromley and Anderson, 1982; Pickett et al., 2013). Rhinaria are divided into two groups: primary and secondary rhinaria. In total, there are three major types of olfactory sensilla on the antennae, consisting of placoid sensilla, coeloconic sensilla, and trichoid sensilla, all of which have been reported in several aphid species, such as *Acyrthosiphon pisum, Megoura viciae, Myzus persicae*, and *Sitobion avenae* (Sun et al., 2013; De Biasio et al., 2015; Bruno et al., 2018; Wu et al., 2022). Primary rhinaria are a group of placoid sensilla and coeloconic sensilla located on the sixth segment and a single large placoid sensillum that occurs on the fifth segment of the antennae (Bromley et al., 1979). Secondary rhinaria contain a large number of small placoid sensilla abundant on the third segment of the antenna of male aphids, more than in females (Bromley et al., 1979; Hardie et al., 1994b; Park and Hardie, 2002). There are two major types of trichoid sensilla: type I trichoid sensilla are distributed on all segments of the antennae, while type II trichoid sensilla are only located on the end of the antennae (Bromley et al., 1980).

Primary rhinaria play an extensive role in the chemoreception of aphids and generally respond to a broad spectrum of volatile semiochemicals (Bromley and Anderson, 1982; Park et al., 2000; Park and Hardie, 2002, 2004; Pope et al., 2004). For example, single sensillum recording (SSR) indicated that the primary rhinaria of currant-lettuce aphid, *Nasonovia ribisnigri*, are responsive to three main classes of chemical compounds: aliphatics, aromatic, and terpenes (Bromley and Anderson, 1982). Another electrophysiological study demonstrated that the placoid sensilla on the fifth antennal segment of primary rhinaria of several aphid species, such as *Aphis fabae, Brevicoryne brassicae*, and *Lipaphis erysimi*, are sensitive to isothiocyanate (Dawson et al., 1987b). Previous studies indicated that the secondary rhinaria are responsible for detecting the aphid sex pheromone (Dawson et al., 1987a). Electrophysiological studies demonstrated that the secondary rhinaria of *M. viciae, Sitobion avenae*, and *Brevicoryne brassicae* were responsive to nepetalactone (Dawson et al., 1987a; Lilley et al., 1994; Gabryś et al., 1997). Additionally, the secondary rhinaria of *Phorodon humuli* showed major activity to nepetalactol (Campbell et al., 1990). A recent study demonstrated the molecular mechanism of alarm pheromone detection in aphids and showed that ApisOR5-expressed neurons housed in the large placoid sensilla on the sixth antennal segment are the major sensilla responding to the alarm pheromone *trans*-β-farnesene (EBF) in *A. pisum* (Zhang et al., 2017). Two ORs in *A. pisum* were found to detect plant volatiles. One of them, ApisOR23 was tuned to five common volatiles of plants and could play an important role in host plant detection, while ApisOR20 was involved in detecting herbivore-induced plant volatile (HIPV) *cis*-jasmone (Huang et al., 2022; Wang et al., 2022b).

Although previous studies have reported that the primary rhinaria were mainly responsible for semiochemical detection in several Aphidinae species, little is known about the odorant detection beyond the subfamily Aphidinae. To better understand the mechanism of olfactory recognition in different subfamilies of Aphididae, we selected three aphid species, *Cinara cedri, Eriosoma lanigerum*, and *Therioaphis trifolii*, from the subfamilies Lachninae, Eriosomatinae, and Calaphidinae, respectively, to perform comparative electrophysiological studies of antennal ORN responses to plant volatiles. In this study, the morphology of the antennal sensilla of *C. cedri, E. lanigerum*, and *T. trifolii* was observed by scanning electron microscopy. Then, the neuronal function of the antennal sensilla in the primary rhinaria was recorded by single sensillum recording (SSR) technology. The neuronal response profile of three aphid species to 18 plant-derived volatiles was mapped. Additionally, the neuronal functions of placoid sensilla in the primary rhinaria were compared based on sensillum type and aphid species. Our results preliminarily illustrate the neuronal mechanisms of olfactory detection at the peripheral nervous level in three aphid species, beyond Aphidinae, to plant volatiles.

## Materials and methods

### Insects

Adult *C. cedri* was collected from *Cedrus deodara* (Roxb.) in the Institute of Plant Protection, Chinese Academy of Agricultural Sciences (CAAS), Beijing, China (39°90′N, 116°30′E). *T. trifolii* was collected from the alfalfa fields at the Langfang Experiment Station of the Institute of Plant Protection, Chinese Academy of Agricultural Sciences (CAAS), Langfang, Hebei province, China (39°50′N, 116°60′E), and reared in our laboratory at 20–22°C, 60–70% relative humidity, and under a photoperiod of 16:8 h (light: dark). *E. Lanigerum* was collected from *Malus pumila* in the suburbs of Tianjin, China (38°95′N, 116°97′E). Adult apterous viviparae were used in all experiments.

### Scanning electron microscopy

Antennae of aphids were excised from the base with fine forceps and dehydrated in ethanol serial solutions (70, 80, 90, and 100%). After drying in a Critical Point Dryer (LEICA CPD 030, Wetzlar, Germany), the samples were coated in gold by an ion sputtering device (HITACHI MC 1000, Tokyo, Japan). Finally, the samples were examined with a HITACHI SU8010 scanning electron microscope (Hitachi, Tokyo, Japan) at 3–10 kV. Pictures were only adjusted for brightness and contrast using Adobe Photoshop CS6 (Adobe Systems). Sensilla types were classified according to methods used by previous studies (Bromley et al., 1979; Bromley and Anderson, 1982; Kanturski et al., 2017).

### Single sensillum recording

A single aphid was stuck to a coverslip with double-face adhesive tape (Zhang et al., 2017). Then the aphid was placed under a LEICA Z16 APO microscope and the antenna was viewed at 920× magnification. Two tungsten microelectrodes were electrolytically sharpened in a 40% KNO_2_ solution. The reference electrode, which was connected to the ground, was inserted into the abdomen of the aphid. The recording electrode was inserted into the plate of the sensillum and connected to the preamplifier (10×, Syntech, Kirchzarten, Germany). An electronic circuit was established by these electrodes to extracellularly record ORN action potentials (Pellegrino et al., 2010). An analog-digital converter (IDAC-4, Syntech, Germany) was connected to the preamplifier and then sent signals to a computer for recording and visualization.

### Odor stimulation

In total, 18 volatile plant compounds with different chemical structures were chosen for the study (Table 1). Common volatiles of host plants of aphids, such as α-terpinene, α-pinene, (–)-β-pinene, sabinene, and (+)-limonene, were included in the selection. Another compound, (*E*)-2-hexenal, belongs to the green leaf volatiles and is one of the most abundant volatiles found in numerous plants. Methyl salicylate, on the other hand, is a known HIPV. In addition, three compounds, namely nepetalactol, nepetalactone, and allyl isothiocyanate, are associated with non-host-plants of the tested aphids. The study aims to determine the function of ORNs from three aphid species in response to compounds from different sources.

**Table 1:** Chemicals used for single sensillum recording.

**Stimulus compounds**	**CAS number**	**Purity (%)**	**Company**
**Monoterpenes**			
α-Terpinene	99-86-5	95	Sigma
α-Pinene	80-56-8	98	Sigma
(–)-β-Pinene	18172-67-3	99	Sigma
Sabinene	3387-41-5	75	Sigma
(+)-Limonene	5989-27-5	97	Sigma
**Monoterpenoids**			
Citral	5392-40-5	>96	Sigma
(–)-Myrtenal	18486-69-6	98	Sigma
(±)-Citronellal	106-23-0	>95	Sigma
β-Citronellol	106-22-9	95	Sigma
(–)-Linalool	126-91-0	97	Sigma
α-Terpineol	98-55-5	>96	Sigma
Nepetalactol	109215-55-6	98	J and K Scientific
Nepetalactone	490-10-8	>98	Bioberry
(–)-Piperitone	4573-50-6	94	Sigma
Geranyl acetate	105-87-3	97	Sigma
**Aromatic**			
Methyl salicylate	119-36-8	>99	Sigma
**Aliphatic compounds and the derivatives**			
*trans*-2-hexenal	6728-26-3	>95	Sigma
Allyl isothiocyanate	57-06-25	>95	ChromaDex

The tested chemicals (94–99% minimum purity) were purchased from Sigma-Aldrich Co. (St. Louis, MO, United States) and used for single sensillum recording (Table 1). Each of the chemicals was diluted in paraffin oil to a stock solution with a concentration of 1 mg/μL. Subsequently, a series of 10-fold dilutions were prepared. For stimulus delivery, 10 μL of each solution was dispersed onto a filter paper strip (0.5 × 3.5 cm), which was then placed into a Pasteur pipette. In the dose-dependent manner, chemicals were tested at a range of dose from 10^−7^ to 10^−3^ g, respectively. A sample containing only paraffin oil served as a control, tested at the beginning and at the end of each recording (Dong et al., 2021; Wang et al., 2022a). The order of the stimuli was randomized, and the time between two consecutive stimuli was 60 s. An airflow maintained at constant 1.2 L/min across an aluminum tube (10-mm inner diameter) was delivered to the antennae throughout the experiment. The distance between the end of the aluminum tube and the antennae was ~1 cm. There was a small hole 10 cm away from the end of the aluminum tube into which the tip of the Pasteur pipette could be inserted. Tested odorants flowed into the air stream (0.5 L/min) through the hole and were then delivered to the antennal sensilla for 300 ms using a Syntech Stimulus controller (CS-55 model, Syntech). For most of the sampled responses, the action potentials (spikes of all sizes) of ORNs in each sensillum were counted offline over a 1 s period (500 ms before and 500 after stimulation onset) by the software package Autospike v. 3.9 (Syntech, Kirchzarten, Germany) (Liu et al., 2013; Dong et al., 2021; Wang et al., 2022a). To accurately assess the spikes generated by chemicals, we plotted the difference of the number of action potentials fired after minus before the stimulus onset.

### Statistical analysis

Graphs and statistics about sensilla responses and dose responses were generated by GraphPad Prism 7.0 (GraphPad Software, La Jolla, CA). All data were calculated as mean ± SEM. A heat map was generated in HemI 1.0 (Deng et al., 2014). All statistical comparisons were performed by SPSS 22.0 (SPSS Inc., Chicago, IL). Two-sample analysis was performed using Student's *t-*test (α = 0.05). Multiple comparisons of data were assessed by one-way analysis of variance (ANOVA) following Duncan's multiple range test (α = 0.05).

## Results

### Antennal shape in three aphid species

**Figure 1:**
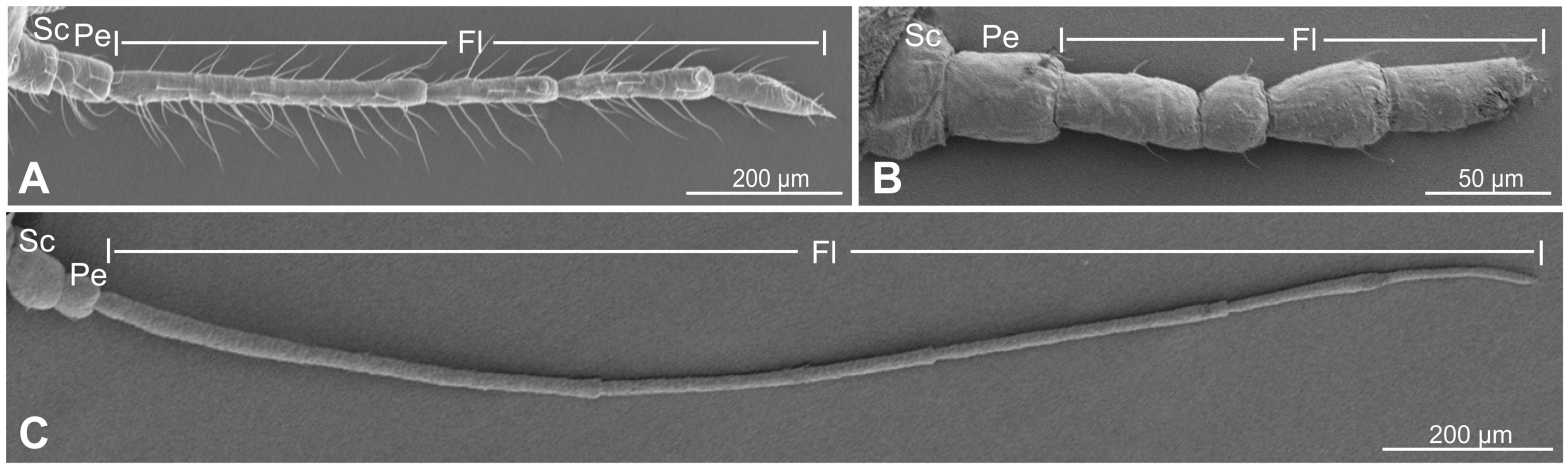
Full views of the antenna in three aphid species. **(A)** *Cinara cedri*; **(B)** *Eriosoma lanigerum*; **(C)** *T. trifolii*. Sc, scape; Pe, pedicel; Fl, flagellum.

Scanning electron microscopy (SEM) observation revealed that the antennae of *C. cedri, Eriosoma lanigerum*, and *Therioaphis trifolii* were all composed of three parts: the scape (Sc), pedicel (Pe), and flagellum (Fl) (Figures 1A–C). The total antennal length of the three aphid species was 9.44 ± 0.44 mm, 3.13 ± 0.18 mm, and 17.95 ± 0.48 mm, respectively. The antennae of *T. trifolii* were comparatively more slender and elongated than those of *E. lanigerum* and *C. cedri*, and the antenna of *E. lanigerum* was stocky and shortest of all three species. *C. cedr*i had a greater density of trichoid sensilla than *T. trifolii* and *E. lanigerum*.

### Identification of antennal sensilla in *Cinara cedri*

**Figure 2:**
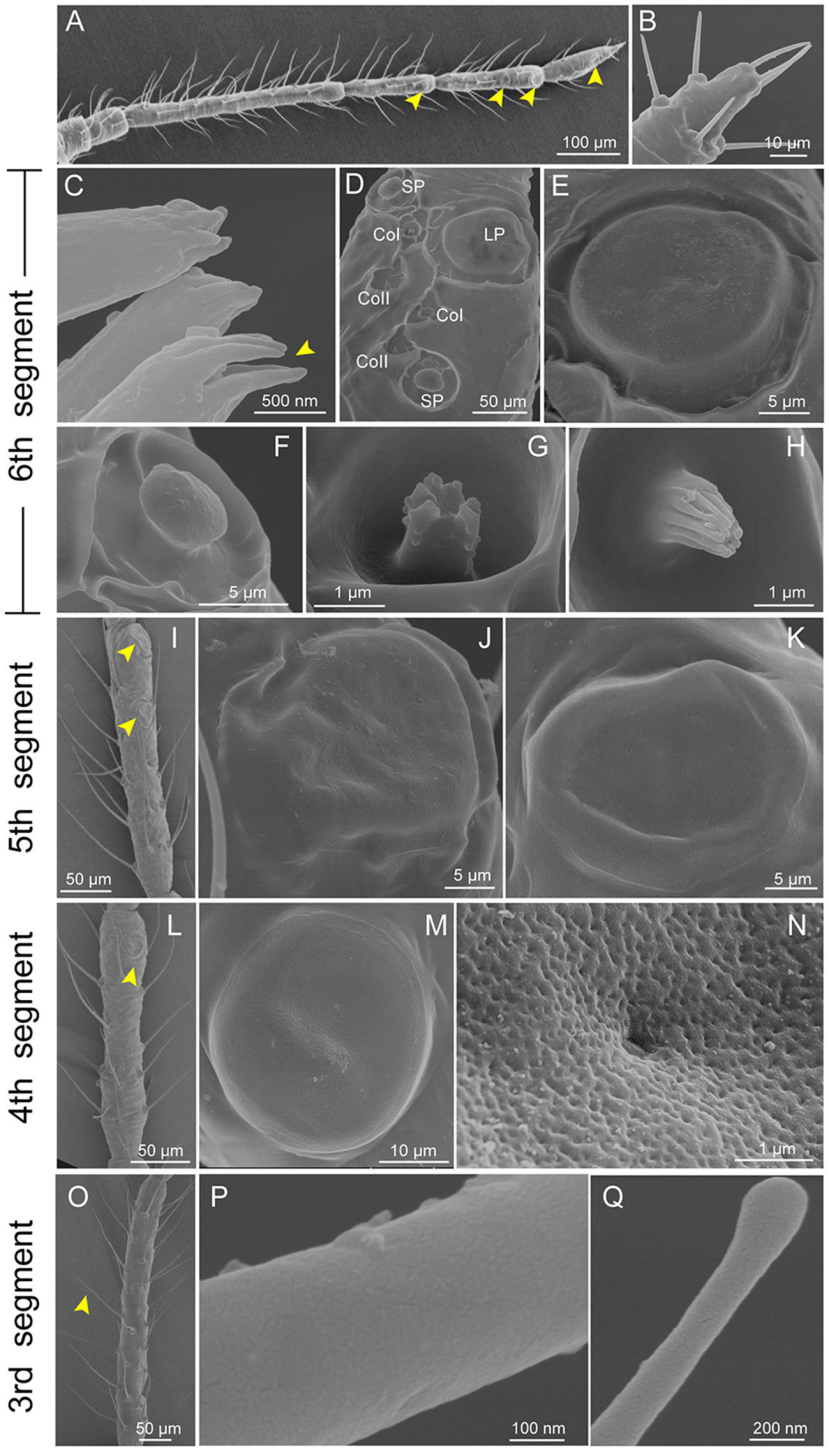
Scanning electron micrographs of *Cinara cedri* antenna. **(A)** A whole view of the antenna shows the primary rhinaria (yellow arrowheads). **(B)** Type II trichoid sensilla at the distal end of the antennae. **(C)** Type II trichoid sensilla with fissure-like structures on the surface with no grooves and had a pore on the top center (yellow arrowheads). **(D)** The primary rhinarium on the 6th segment consists of one large placoid sensillum (LP), two small placoid sensilla (SP), and four coeloconic sensilla (2 CoI and 2 CoII). **(E–H)** An enlarged view of the LP **(E)**, SP **(F)**, CoI pegs **(G)**, and CoII pegs **(H)** on the 6th segment. **(I)** A whole view of the primary rhinarium (yellow arrowheads) on the 5th segment consists of two large placoid sensilla (LP5I and LP5II). **(J, K)** An enlarged view of LP5I and LP5II. **(L)** A whole view of the large placoid sensillum (yellow arrowhead) on the 4th segment (LP4). **(M)** An enlarged view of the LP4. **(N)** A porous surface of LP4. **(O)** Type I trichoid sensilla (arrowhead) located on the 3rd segment. **(P, Q)** Type I trichoid sensilla showing a smooth surface **(P)** and a swollen tip with no pore **(Q)**.

There were six segments in the antennae of *C. cedri* from the distal to the proximal end, showing an obvious primary rhinarium at the distal end (Figure 2A). Three distinct types of sensilla were observed on the entire surface of the antennae, including trichoid sensilla, placoid sensilla, and coeloconic sensilla (Figures 2B–Q). Trichoid sensilla were classified into 2 types based on their morphology. The type I trichoid sensilla was distributed on all segments of the antennae (Figure 2O) and presented a swollen tip with no pores on the surface (Figures 2P, Q). The type II trichoid sensilla with hemispherical sockets were located on the tip region of the 6th antennal segment (Figures 2B, C). These sensilla had fissure-like structures on the surface with no grooves and had a pore on the top center of the claw-like structures (Figure 2C).

Primary rhinaria are present on the 4th, 5th, and 6th segments in *C. cedri*, but only on the 5th and 6th segments in the other aphid species (Song et al., 2020). The primary rhinarium on the 6th segment was composed of one large placoid sensillum (LP6), two small placoid sensilla (SP6), and four coeloconic sensilla (Figures 2D–H). The primary rhinarium on the 5th segment consisted of two large placoid sensilla, type I (LP5I) and type II (LP5II) (Figures 2I–K). The primary rhinarium on the 4th segment had a single large placoid sensillum (LP4) (Figures 2L, M). The large placoid sensilla looked like circular plates with slight folds on the surface, situated in a shallow ridge (Figures 2E, J, K, M), while the small placoid sensilla were mushroom-shaped (Figure 2F). All the placoid sensilla were not surrounded by cuticular fringe structures and had smooth surfaces with small pores (Figure 2N).

Coeloconic sensilla were classified into two types, which were typical in a peg-in-pit shape (Figure 2D). Type I coeloconic sensilla exhibited a peg tip with a crown consisting of six cuticular projections (Figure 2G). The tip of the peg of type II coeloconic sensilla gathered like a flower bud, with a varying number of cuticular projections (Figure 2H).

### Identification of antennal sensilla in *Eriosoma lanigerum*

**Figure 3:**
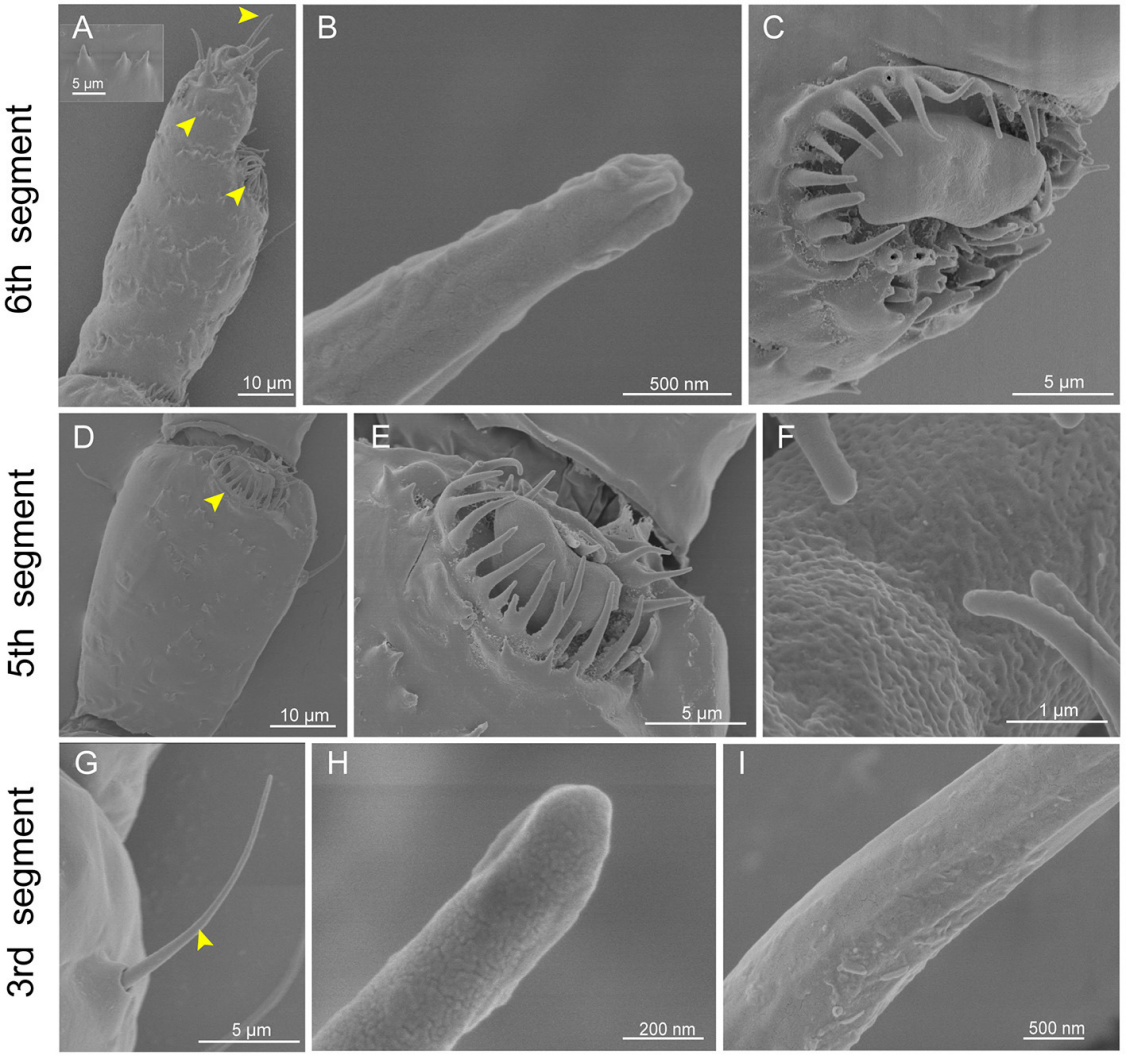
Scanning electron micrographs of *Eriosoma lanigerum* antennae. **(A)** A view of the 6th segment of the primary rhinarium (arrowhead on right), type II trichoid sensilla (arrowhead on top), and spikeweed-like protrusions on the surface (arrowhead on left). **(B)** An enlarged view of type II trichoid sensilla showing a finger-like pore slit on the tip. **(C)** An enlarged view of the large placoid sensillum of the primary rhinarium on the 6th segment (LP6). **(D)** The primary rhinarium on the 5th segment consists of one large placoid sensillum (LP5) (yellow arrowheads). **(E)** An enlarged view of LP5. **(F)** LP5 with a porous surface. **(G)** Type I trichoid sensilla (yellow arrowhead) located on the 3rd segment. **(H, I)** Details of type I trichoid sensilla showing a smooth surface **(I)** with no pore structures on the tip **(H)**.

Six segments of the antennae were observed in *E. lanigerum*, and the primary rhinaria are located on the 5th and 6th segments (Figure 1B). In the 6th segment, there were several type II trichoid sensilla on the distal end of the antennae (Figure 3A). The shape of type II trichoid sensilla was similar to that of *C. cedri* (Figure 3B). Additionally, an LP6 on the 6th segment and an LP5 on the 5th segment of the primary rhinaria were observed (Figures 3A, C–E). LP6 and LP5 were both in the form of a flat plate with many pores located on the surface (Figure 3F), and surrounded by the cuticular ridge (Figures 3C, E). There were few type I trichoid sensilla on the whole antennae (Figure 1B). This sensilla looks like a long hair with a grooved surface and had a blunt and swollen poreless tip (Figures 3G–I). Compared to other aphids, there was a spikeweed-like protrusion structure located on the surface of the *E. lanigerum* antenna (Figure 3A).

### Olfactory response profiles of three aphid species

**Figure 4:**
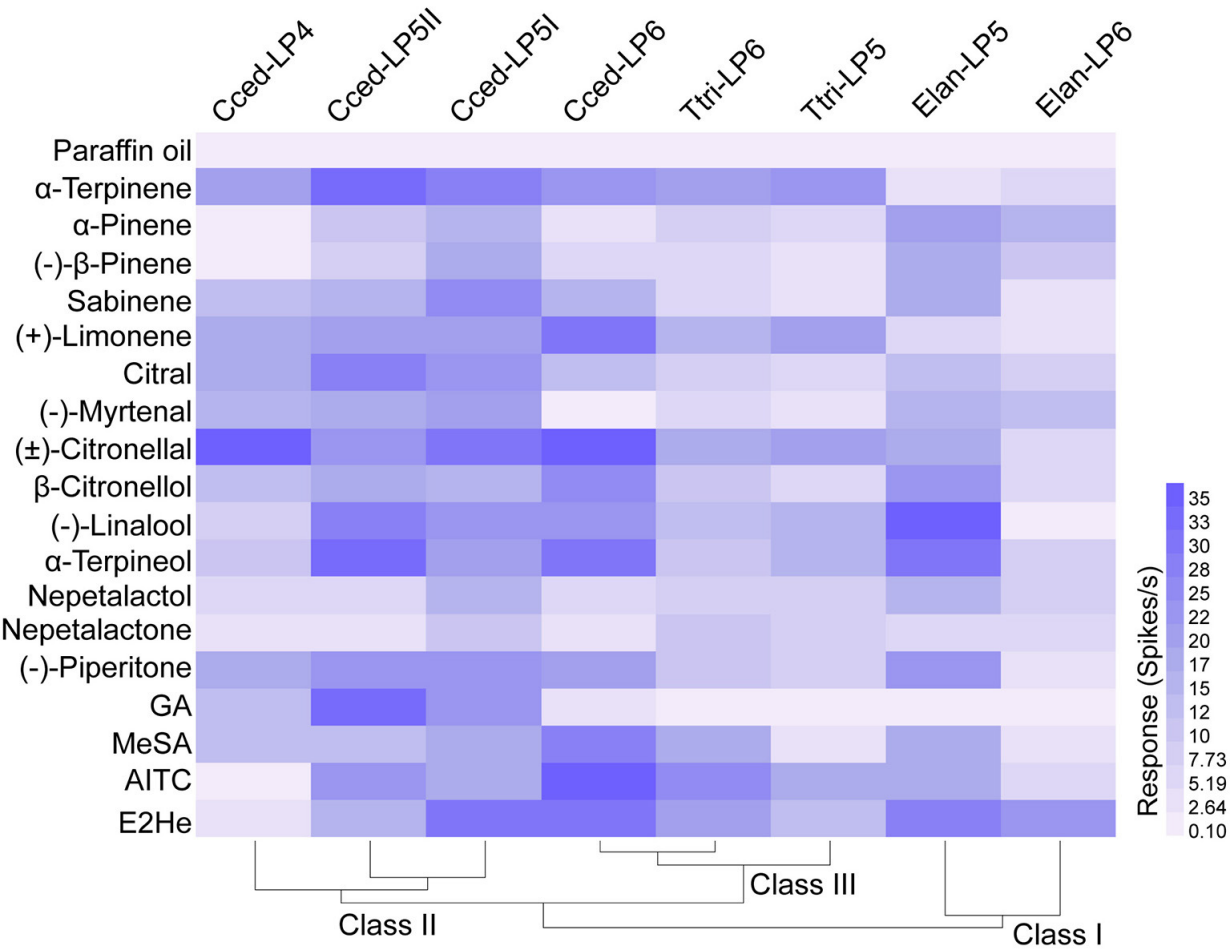
Heat map of functional profiles of ORNs housed in primary rhinaria in *C. cedri, E. lanigerum*, and *T. trifolii* to the tested odorants. The response intensity of ORNs is color-coded according to the response values (*n* = 4–16). The solvent paraffin oil was set as the control. Chemicals were listed in Table 1. E2He, *trans*-2-hexenal; MeSA, methyl salicylate; AITC, allyl isothiocyanate, GA, geranyl acetate.

Based on the SEM observation of primary rhinaria in *C. cedri* and *E. lanigerum*, and previous reports about similar observations in *T. trifolii* (Song et al., 2020), we conducted functional characterizations of ORNs housed in the placoid sensilla of primary rhinaria and how they responded to 18 chemicals derived from plant volatiles (Table 1). Functional profiles of primary rhinaria in the three aphid species were divided into three classes. Functional class I consisted of neuronal responses of LP6 and LP5 in *E. lanigerum*, class II was found in LP5I, LP5II, and LP4 in *C. cedri*, and class III contained similar responses between LP6 and LP5 in *T. trifolii* and LP6 in *C. cedri* (Figure 4). The clustering results showed that neuronal responses generally exhibited a species-specific pattern. However, in *C. cedri*, the neuronal function of LP6 differed from that of the other three sensilla, indicating functional differentiation has occurred in primary rhinaria of *C. cedri*.

### Neuronal response of primary rhinaria in *C. cedri* to plant-derived volatiles

**Figure 5:**
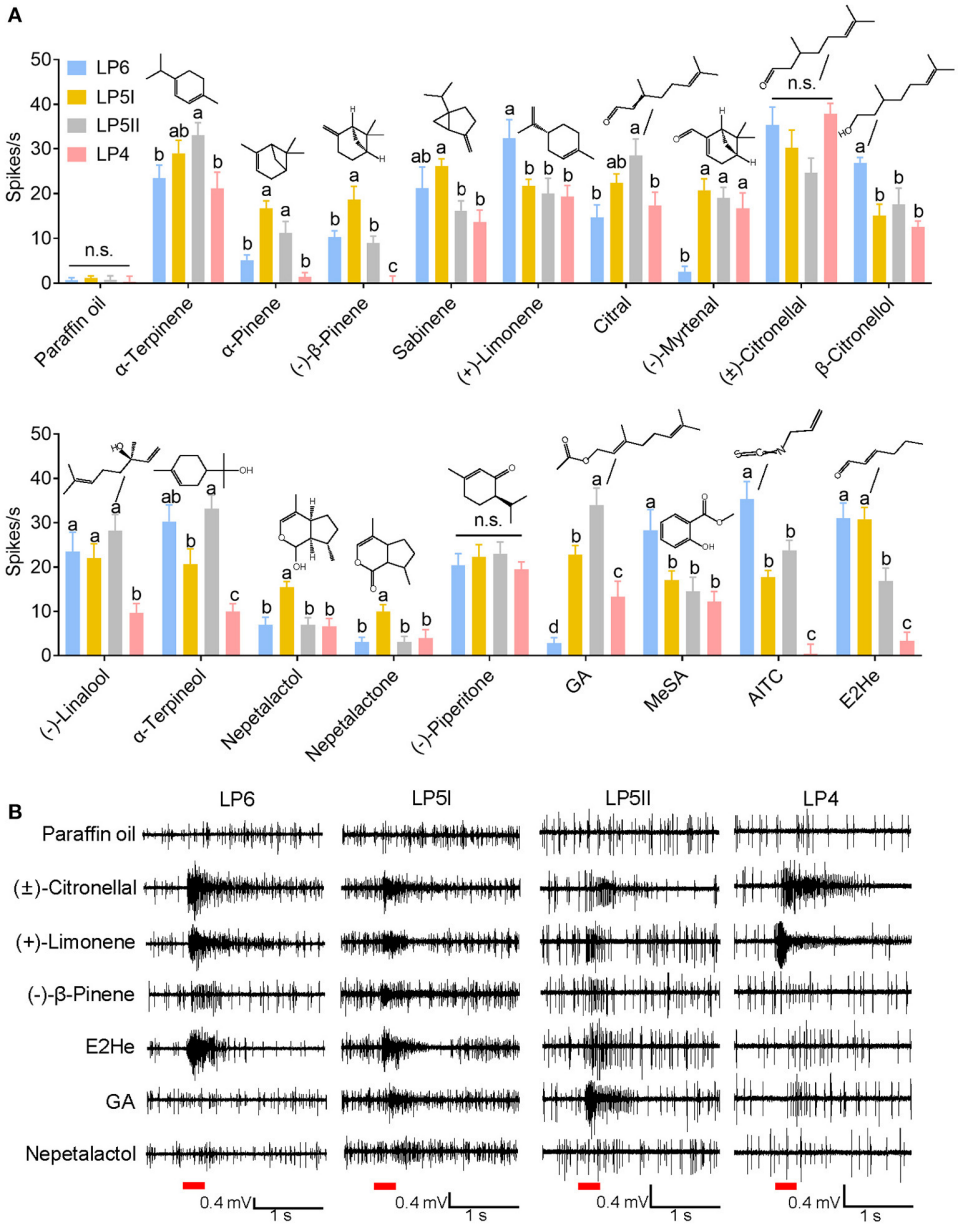
SSR response values of ORNs in antennal placoid sensilla of *C. cedri* to the tested odorants. **(A)** Neuronal responses of LP4, LP5I, LP5II, and LP6 of *C. cedri*. Bars labeled with different letters are significantly different (mean ± SEM, *n* = 4–16, GLM followed by Duncan's multiple range test). **(B)** Representative SSR traces showing responses of ORNs in LP4, LP5I, LP5II, and LP6 of *C. cedri* to (±)-citronellal, (+)-limonene, (–)-β-pinene, E2He, GA, and nepetalactol. Paraffin oil was used as a control. Chemicals were tested at a dose of 10^−3^ g. Red bars indicate the 300 ms stimulation of odorants. E2He, *trans*-2-hexenal; MeSA, methyl salicylate; AITC, allyl isothiocyanate; GA, geranyl acetate.

**Figure 6:**
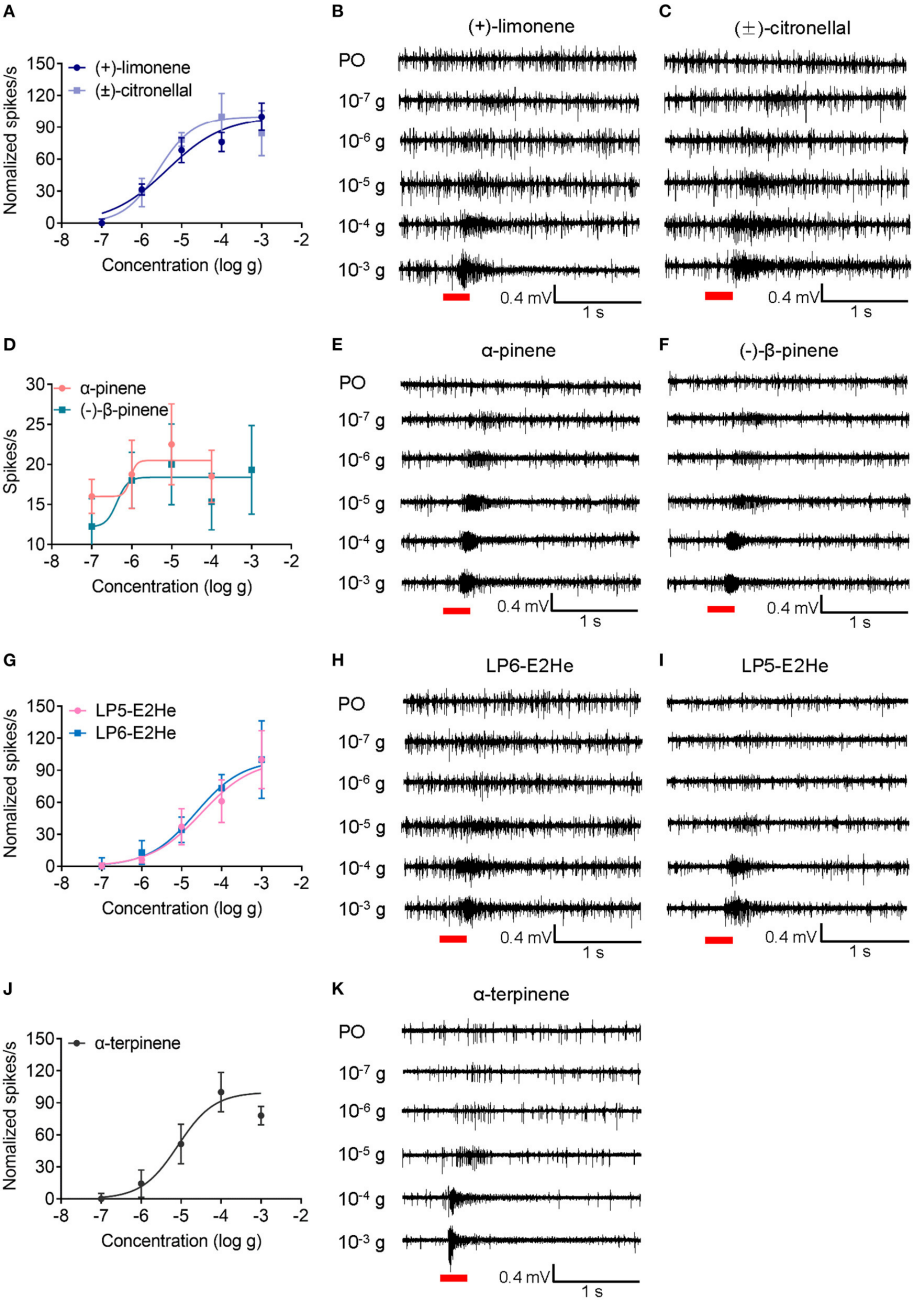
Excitatory dose-dependent responses of ORNs in placoid sensilla of *C. cedri* to the tested odorants. **(A)** Dose-response curves of ORNs in LP6 to (+)-limonene and (±)-citronellal across a range of doses from 10^−7^ to 10^−3^ g. The EC_50_ values induced by (+)-limonene and (±)-citronellal are 4.31 × 10^−6^ g (*n* = 4) and 2.62 × 10^−6^ g (*n* = 4), respectively. **(B, C)** SSR traces showing ORNs were activated by (+)-limonene and (±)-citronellal. **(D)** Dose-response curves of ORNs in LP5I to α-pinene and (–)-β-pinene with EC_50_ values of 1.10 × 10^−6^ g (*n* = 4) and 8.40 × 10^−7^ g (*n* = 4), respectively. **(E, F)** SSR traces showing ORNs were activated by α-pinene and (–)-β-pinene. **(G)** Dose-response curves of ORNs in LP5I and LP6 to *trans*-2-hexenal with EC_50_ values of 3.13 × 10^−5^ g (*n* = 4) and 2.24 × 10^−5^ g (*n* = 4), respectively. **(H, I)** SSR traces showing ORNs in LP6 and LP5I were activated by *trans*-2-hexenal. **(J)** Dose-response curves of ORNs in LP5II to α-terpinene with the EC_50_ value of 8.36 × 10^−6^ g (*n* = 4). **(K)** SSR traces showing ORNs in LP5II were activated by α-terpinene. Paraffin oil (PO) was used as a control. Chemicals were tested at a dose of 10^−7^, 10^−6^, 10^−5^, 10^−4^, and 10^−3^ g, respectively. Error bars indicate SEM. Red bars indicate the 300 ms stimulation of odorants.

Four distinct placoid sensilla (LP6, LP5I, LP5II, and LP4) in the primary rhinaria of *C. cedri* were tested using 18 chemicals. Neuronal responses in all tested placoid sensilla were activated by (±)-citronellal and (–)-piperitone with no significant differences observed (*P* > 0.05) (Figure 5). The ORNs in LP6 showed excitatory responses to all tested chemicals except (–)-myrtenal, geranyl acetate, and nepetalactone (<5 spikes/s), the highest response of which was induced by (±)-citronellal (35.33 ± 4.08 spikes/s). In particular, ORNs in LP6 significantly responded to (+)-limonene (32.3 ± 4.2 spikes/s), allyl isothiocyanate (AITC, 35.3 ± 4.0 spikes/s), methyl salicylate (28.3 ± 4.6 spikes/s), and β-citronellol (26.8 ± 1.2 spikes/s) compared to neuronal responses in the other three sensilla (*P* < 0.05). Furthermore, neuronal responses in LP6 to (+)-limonene and (±)-citronellal both showed dose-dependent patterns at a range of dose from 10^−7^ to 10^−3^ g, with EC_50_ values of 4.31 × 10^−6^ g and 2.62 × 10^−6^ g, respectively (Figures 6A–C).

In the LP5I sensillum, ORNs strongly responded to *trans*-2-hexenal (30.8 ± 2.6 spikes/s), (±)-citronellal (30.2 ± 3.9 spikes/s), and α-terpinene (29.0 ± 2.9 spikes/s), but were inclined to α-pinene (16.8 ± 1.6 spikes/s), (–)-β-pinene (18.7 ± 2.9 spikes/s), nepetalactol (15.5 ± 1.2 spikes/s), and nepetalactone (10.0 ± 1.5 spikes/s) compared to the other three sensilla (*P* < 0.05). We recorded the responses of ORNs in LP5I to α-pinene and (–)-β-pinene at a range of dose from 10^−7^ to 10^−3^ g. The results demonstrated that ORNs were more sensitive to (–)-β-pinene (EC_50_ = 8.40 × 10^−7^ g) than to α-pinene (EC_50_ = 1.10 × 10^−6^ g) (Figures 6D–F). We also compared the sensitivities of ORNs in LP6 and LP5I to *trans*-2-hexenal. The dose-dependent responses of ORNs in LP6 (EC_50_ = 2.24 × 10^−5^ g) showed similar sensitivity to that in LP5I (EC_50_ = 3.13 × 10^−5^ g) (Figures 6G–I).

The ORNs in LP5II showed excitatory responses to α-terpineol (33.2 ± 3.0 spikes/s), α-terpinene (33.0 ± 2.8 spikes/s), and geranyl acetate (34.0 ± 3.8 spikes/s). The response of neurons in LP5II to α-terpinene also exhibited a dose-dependent pattern (EC_50_ = 8.36 × 10^−6^ g). As the dose increased from 10^−7^ to 10^−3^ g, the neuronal firing rate increased from 6.5 to 28 spikes/s, while the neuronal firing rate decreased to 24.25 spikes/s at a higher dose of 10^−3^ g (Figures 6J, K). The ORNs in LP4 sensillum had the weakest response to most of the chemicals among the four types of the placoid sensilla, except for (±)-citronellal (37.8 ± 2.5 spikes/s) (Figures 4, 5).

### Neuronal response of primary rhinaria in *E. lanigerum* and *T. trifolii* to plant-derived volatiles

**Figure 7:**
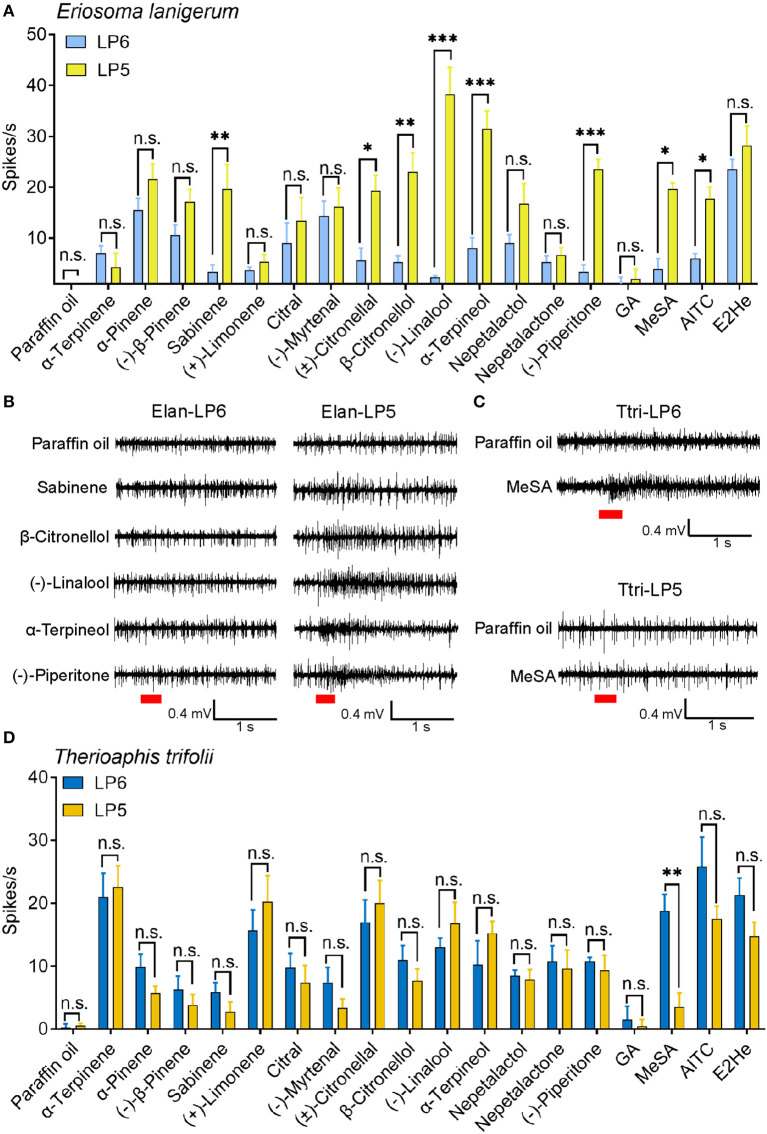
SSR response values of ORNs in antennal placoid sensilla of *E. lanigerum* and *T. trifolii* to the tested odorants. **(A)** Neuronal responses of LP5 and LP6 of *E. lanigerum* (*n* = 3–10). Error bars indicate SEM (Student's *t*-test, *P < 0.05, **P < 0.01, ***P < 0.001, n.s. represents no significant differences). **(B)** Neuronal responses of LP5 and LP6 of *T. trifolii* (*n* = 3–11). **(C)** The representative SSR traces showing ORNs in LP6 and LP5 of *E. lanigerum* were tested by sabinene, β-citronellol, (–)-linalool, α-terpineol and (–)-piperitone. **(D)** Representative SSR traces showing responses of ORNs in LP6 and LP5 of *T. trifolii* to MeSA. Paraffin oil was used as a control. Chemicals were tested at a dose of 10^−3^ g. Red bars indicate the 300 ms stimulation of odorants. E2He, *trans*-2-hexenal; MeSA, methyl salicylate; AITC, allyl isothiocyanate; GA, geranyl acetate.

In *E. Lanigerum*, most of the tested chemicals elicited larger responses from ORNs in LP5 than in LP6 (Figures 4, 7A). In particular, ORNs in LP5 were more strongly activated by (–)-linalool (38.3 ± 5.3 spikes/s), α-terpineol (31.5 ± 3.5 spikes/s), and (–)-piperitone (23.5 ± 1.9 spikes/s) compared to that in LP6 (*P* < 0.001). β-citronellol, sabinene, methyl salicylate, (±)-citronellal, and AITC also activated ORNs significantly more in LP5 than in LP6 with mean values of 23.0 ± 3.8 (*P* < 0.01), 21.3 ± 3.2 (*P* < 0.01), 19.7 ± 1.2 (*P* < 0.05), 19.3 ± 3.1 (*P* < 0.05), and 17.7 ± 2.3 spikes/s (*P* < 0.05) respectively. However, ORN responses to the remaining components showed no difference between LP5 and LP6 (*P* > 0.05) (Figures 7A, B).

In *T. trifolii*, methyl salicylate significantly elicited higher responses in ORNs in LP6 (18.8 ± 2.7 spikes/s) than in LP5 ORNs (3.5 ± 2.2 spikes/s) (*P* < 0.01), while responses of ORNs to other components did not show any significant differences between LP5 and LP6 (*P* > 0.05) (Figures 7C, D). Moreover, ORNs in LP6 responded to most tested odorants except geranyl acetate (<5 spikes/s) and exhibited strong responses to AITC (25.8 ± 4.7 spikes/s), *trans*-2-hexenal (21.3 ± 2.7 spikes/s), α-terpinene (21.0 ± 3.8 spikes/s), and (±)-citronellal (17.0 ± 3.5 spikes/s). ORNs in LP5 mainly responded to α-terpinene, (+)-limonene, (±)-citronellal, AITC, and (-)-linalool, which elicited firing frequencies of 22.6 ± 2.4, 20.3 ± 4.2, 20.0 ± 3.6, 17.5 ± 2.0, and 16.8 ± 3.4 spikes/s, respectively (Figure 7D).

### Comparison of the neuronal responses in LP5 and LP6 among three aphid species

**Figure 8:**
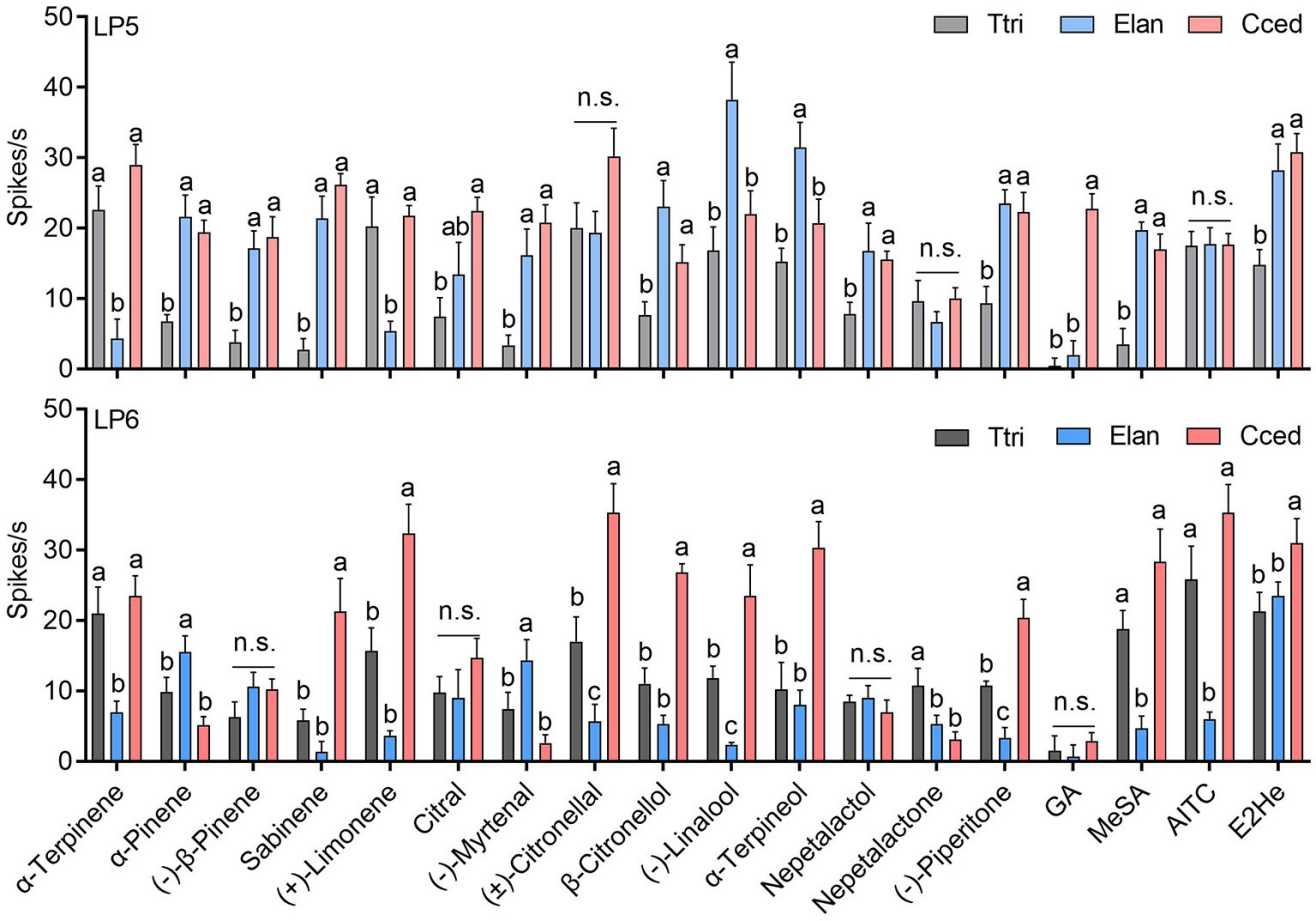
Comparison of the neuronal response values between LP5 and LP6 among *C. cedri, E. lanigerum*, and *T. trifolii* to the tested odorants. Bars labeled with different letters are significantly different; n.s. indicates no significant differences (mean ± SEM, *n* = 3–13, GLM followed by Duncan's multiple range test). E2He, *trans*-2-hexenal; MeSA, methyl salicylate; AITC, allyl isothiocyanate; GA, geranyl acetate.

Functional characterization of ORNs in LP5 and LP6 sensilla were compared among three aphid species. In general, neuronal activities in LP5 of *T. trifolii* to 9 tested chemicals (e.g., *trans*-2-hexenal and methyl salicylate, among others) were lower than in *C. cedri* and *E. lanigerum* (*P* < 0.05) (Figure 8). Additionally, ORNs of *E. lanigerum* produced the largest responses to (–)-linalool and α-terpineol compared to *C. cedri* and *T. trifolii*, while *C. cedri* had the strongest response to geranyl acetate compared to *T. trifolii* and *E. lanigerum* (*P* < 0.05). There were no significant differences in neuronal responses among *C. cedri, T. trifolii*, and *E. lanigerum* induced by AITC, (±)-citronellal, and nepetalactone (*P* > 0.05) (Figure 8).

Unlike the functional profiles of LP5, ORNs in LP6 of *C. cedri* have stronger activities compared to the other two species in most cases, especially in response to *trans*-2-hexenal, (–)-piperitone, α-terpineol, (–)-linalool, β-citronellol, (±)-citronellal, (+)-limonene, and sabinene (*P* < 0.05) (Figures 4, 8). However, two exceptions were found. ORNs in *E. lanigerum* showed the highest responses to (–)-myrtenal and α-pinene (*P* < 0.05), while nepetalactone elicited the strongest responses in *T. trifolii* (*P* < 0.05) (Figure 8).

## Discussion

Identifying the antennal structure and sensilla types of aphid species is needed to understand olfactory perception mechanisms in aphids. In this study, we observed and compared the morphology, distribution, and function of the antennal sensilla of three aphid species (*C. cedri, E. lanigerum*, and *T. trifolii*) from three subfamilies of Aphididae, which suggests a role of olfactory detection in the host-plant selection.

Antennae in most aphid species typically contain six segments, including a scape, a pedicel, and a flagellum with 4 flagellomeres (Song et al., 2020; Wu et al., 2022). In this study, six segments were observed in *C. cedri, E. lanigerum*, and *T. trifolii*, but exhibited various lengths, thicknesses, and shapes. However, *Pseudessigella brachychaeta* showed only 3 flagellomeres in the flagellum on the antennae (Kanturski et al., 2017). This indicates that the morphology of the antennae varies between aphid species.

The flagellum has important olfactory functions and typically contains various sensory sensilla in the rhinaria. The primary rhinaria are an important sensory organ of the antennal peripheral nervous system in aphids and are sensitive to a broad spectrum of plant volatiles and alarm pheromones (Bromley and Anderson, 1982; Park et al., 2000; Park and Hardie, 2002, 2004; Pope et al., 2004; Zhang et al., 2017). Usually, a large placoid sensillum on the 5th antennal segment and a group of sensilla (consisting of 1 LP, 2 SPs, and several coeloconic sensilla) on the 6th antennal segment form the primary rhinaria of the antenna in aphids, which is similar to *E. lanigerum* and has been previously reported in *T. trifolii* (Song et al., 2020). However, the composition of primary rhinaria in *C. cedri* presents a new pattern, which consists of 1 LP on the 4th antennal segment, 2 LPs on the 5th antennal segment, and a group of sensilla (consisting of 1 disc-shaped LP, 2 mushroom-shaped SPs, and 4 coeloconic sensilla) on the 6th antennal segment. The shape of SPs on the 6th antennal segments varied in different aphid species. For example, SPs on the 6th antennal segments of *T. trifolii* were replaced by 2 stellate sensilla, which have only been identified in the Drepanosiphinae subfamily of aphids (Shambaugh et al., 1978; Song et al., 2020). Moreover, the results demonstrate that the structure of cuticular fringes surrounded all sensilla of the primary rhinaria in *E. lanigerum* and *T. trifolii*, while they were absent in the sensilla of *C. cedri* (Lee et al., 2020; Song et al., 2020). In general, the coeloconic sensilla were sunken pegs with several finger-like projections and were classified as either type I or type II based on the shape of terminal projections. We found 4 coeloconic sensilla in the primary rhinarium on the 6th antennal segment in *C. cedri*, but 2 to 3 coeloconic sensilla were reported in *T. trifolii* (Song et al., 2020). The structure of these sensilla was similar to those of *M. persicae* and *Megoura viciae* (Ban et al., 2015; Bruno et al., 2018). Multipore structures were found on the smooth surface of sensilla in the primary rhinaria of all three investigated aphid species, suggesting that they are likely involved in the perception of volatiles.

The secondary rhinaria are fewer or absent in the third segment of adult apterous viviparae in *C. cedri, E. lanigerum*, and *T. trifolii* because they are dependent on sexual dimorphism and wing dimorphism. Previous results demonstrated that secondary rhinaria were used to detect sex pheromone components (Dawson et al., 1987a; Pickett et al., 1992). However, it is not known whether they are sensitive to plant volatiles. Two types of trichoid sensilla were observed on the antennae of three aphid species. Of these, type I trichoid sensilla were distributed on the whole antenna surface and had a smooth surface and a swollen poreless tip. Similar morphologies of type I trichoid sensilla were usually observed in some aphid species, such as *A. pisum* (De Biasio et al., 2015) and *M. persicae* (Sun et al., 2013), which could be involved in mechanoreceptive functions (Bromley et al., 1980). In this study, we found that the type I trichoid sensilla had a higher density in *C. cedri* than in *E. lanigerum* and *T. trifolii*, suggesting that the adaptive evolution of sensilla in *C. cedri* could be related to structural features of pine needles. Type II trichoid sensilla were also found on the antennal tip of *C. cedri, E. lanigerum*, and *T. trifolii*, with a blunt tip and a single apical pore, which performed a gustatory function in a previous study (Powell et al., 1995).

To study and compare the roles of the primary rhinaria in three aphid species, we tested the electrophysiological responses of ORNs in the placoid sensilla (primary rhinaria) using 18 plant volatile compounds, including monoterpenes, monoterpenoids, and aliphatics. We found that most of the 18 chemicals could induce neuronal responses in the primary rhinaria of *C. cedri, E. lanigerum*, and *T. trifolii*, but that function varied among species. This demonstrates that ORNs in *C. cedri* are more sensitive to many tested monoterpenes and monoterpenoids, and half of the tested compounds elicited weaker responses in LP5 *T. trifolii* compared to other species. In general, some monoterpenes, such as (+)-limonene, sabinene, α-pinene, and (–)-β-pinene, and monoterpenoids (terpineol) are the major volatiles of the essential oil of *Cedrus* spp. (Gao et al., 2005; Jaouadi et al., 2021), which are host plants of *C. cedri*. We found that four large placoid sensilla of the primary rhinaria in *C. cedri* were all responsive to host plant volatiles, (+)-limonene, terpineol, and sabinene. This demonstrates that the strongest responses of *C. cedri* to monoterpenes and monoterpenoids were due to the detection of host plant volatiles. Moreover, we found that ORNs in the primary rhinaria of all three aphid species were strongly stimulated by the green leaf volatile *trans*-2-hexenal, which is one of the most abundant volatiles of plants (Tava and Pecetti, 1997; Giacomuzzi et al., 2016; Jaouadi et al., 2021).

The responses of ORNs between LP5 and LP6 of the primary rhinaria in three aphid species show functional differences to specific chemicals. For example, AITC is the major component in mustard oil and could elicit neuronal responses in the LP5 of primary rhinaria in *Brevicoryne brassicae* (L.), *A. fabae*, and *A. pisum* (Nottingham et al., 1991; Zhang et al., 2017). We found the same result in LP5 of *E. lanigerum*, but obtained different results in *C. cedri*, demonstrating that AITC elicited a significantly greater neuronal response in LP6 than in LP5. In *T. trifolii*, in contrast to LP5, ORNs in LP6 are strongly responding to methyl salicylate, which is a volatile emitted by herbivores-damaged *Trifolium pratense* (Kigathi et al., 2009). This indicates that ORNs in LP6 of *T. trifolii* are important for detecting plant secondary metabolites, and could be involved in the behavioral host plant selection of *T. trifolii*. In *E. lanigerum*, the strongest response in LP5 was elicited by (–)-linalool, which is a plant volatile released from both intact and caterpillar-damaged apple foliage (Bengtsson et al., 2001; Giacomuzzi et al., 2016). Some studies indicated that linalool is a key chemical in herbivore deterrence (Borrero-Echeverry et al., 2015; Hatano et al., 2015), however, it is unclear whether it plays the same role in *E. lanigerum*. These electrophysiological results showed that different aphid species had diverse sensitivities to some plant volatile compounds, suggesting this could reflect distinct host-plant requirements (Nottingham et al., 1991).

In general, neuronal responses in the secondary rhinaria of male and gynoparae aphids are sensitive to the well-known sex pheromones of several aphid species, such as *Aphis fabae* and *Rhopalosiphum padi* (L.), while the neuronal activities in the primary rhinaria broadly respond to both plant volatiles and sex pheromone components (Dawson et al., 1987a; Park and Hardie, 2002, 2004; Birkett and Pickett, 2003). In this study, we found that ORNs in the primary rhinaria of adult apterous viviparae in three aphid species could also detect nepetalactone and nepetalactol. We hypothesize that the two compounds that stimulated the primary rhinaria may have originated from plants, as they have been identified as the main constituents of catmint essential oils in previous studies (Zomorodian et al., 2012). Although the primary host of these aphids is not known to contain these compounds, their ability to detect them implies that they may be present in their natural environment. Further investigations are required to confirm this hypothesis.

In conclusion, the antennal sensilla on the primary rhinaria of *C. cedri, E. lanigerum*, and *T. trifolii* from three subfamilies of Aphididae were identified and functionally characterized. The response profiles of ORNs in the placoid sensilla of primary rhinaria respond to plant-derived volatiles and reveal the intraspecific and interspecific variation of neuronal functions in aphids. This result provides a good basis to compare inter-species olfactory detection and host plant selection in different aphid species.

## Data availability statement

The original contributions presented in the study are included in the article, further inquiries can be directed to the corresponding authors.

## Author contributions

G-RW, BW, and L-LY contributed to conception and design of the study and wrote the first draft of the manuscript. L-LY performed the experiments. BW and L-LY performed the statistical analysis. G-RW, BW, JS, and L-LY wrote sections of the manuscript. All authors contributed to manuscript revision, read, and approved the submitted version.
